# The effect of mood on food versus non-food interference among females who are high and low on emotional eating

**DOI:** 10.1186/s40337-021-00497-3

**Published:** 2021-10-29

**Authors:** Hilla Sambal, Cara Bohon, Noam Weinbach

**Affiliations:** 1 School of Psychological Sciences, University of Haifa, Abba Khoushy Ave 199, 3498838 Haifa, Israel; 2 Department of Psychiatry and Behavioral Sciences, Stanford University School of Medicine, Stanford, CA USA

**Keywords:** Emotional eating, Interference control, Attentional bias, Flanker task, Inhibitory control, Mood induction

## Abstract

**Background:**

Emotional eating refers to overeating triggered by emotional experiences and may cause significant psychological distress and health problems. Thus, it is important to better understand its underlying mechanisms. The study examined if the ability to ignore task-irrelevant information, namely, interference control, is modulated by mood and exposure to food stimuli among females who are high and low on emotional eating.

**Method:**

The study’s sample included 80 women who were high (N = 40) or low (N = 40) on an emotional eating scale. Participants were divided to a negative or neutral mood induction group. Following the mood induction, they completed a food-flanker task that allowed assessing attentional interference caused by food and non-food stimuli separately.

**Results:**

The low emotional eating group had significantly greater food compared to non-food interference, suggesting difficulty at ignoring food stimuli while attending a neutral target. In the high emotional eating group, there was no difference between food and non-food interference. However, higher levels of emotional eating predicted lower levels of food interference.

**Conclusion:**

The pattern of results suggests a food-avoidance attentional tendency among those with higher levels of emotional eating. The mood manipulation did not influence food-related interference in either group. The lack of an effect of mood on food-related interference questions the impact of negative emotions on basic attentional processes among individuals with emotional eating.

**Supplementary Information:**

The online version contains supplementary material available at 10.1186/s40337-021-00497-3.

## Plain english summary

Emotional eating is characterized by overeating following the experience of negative emotions. Emotional eating may cause significant psychological distress and health problems. Thus, it is important to better understand its underlying mechanisms. The study assessed how negative mood influences the ability to ignore the presence of high-calorie food images among females who are high or low on an emotional eating scale. Participants’ mood was manipulated by an autobiographic writing task (half of the participants described negative events and the other half emotionally neutral events). Then, they completed a computer task in which they were required to indicate whether a centrally presented image was of food or a non-food item as fast as possible. Next to the central image, there were additional images that were used for distraction. These images were also images of food or non-food items. Participants in the low emotional eating group showed greater distraction when food items were used for distraction. In contrast, those in the high emotional eating group were not distracted by food items. In fact, higher levels of emotional eating were associated with diversion of attention away from food stimuli, irrespective of mood. We suggest that these results reflect a broader avoidant strategy that is activated in response to emotionally negative content among individuals with emotional eating.

**Supplementary Information:**

The online version contains supplementary material available at 10.1186/s40337-021-00497-3.

## Background

Food intake is not only regulated by physical needs, but is also affected by emotional states, motivations, and self-regulatory processes [1]. Emotional eating (EE) refers to the tendency to eat in response to emotions, most often negative emotions. Since eating can be rewarding and comforting, EE is thought to occur in an effort to regulate negative emotion in the absence of more adaptive coping strategies [2]. Previous studies reported that high levels of EE were associated with weight change and obesity, increased risk for eating disorders, and elevated levels of anxiety and depression [3–7].

Despite its potentially distressing effects, the behavioral and brain mechanisms that drive EE are still unclear. In fact, a recent meta-analysis challenged the basic notion that negative emotions increase food intake among those who identify themselves as high on EE [8]. Specifically, weak effect sizes were reported in laboratory-based studies examining if people who score high on EE scales overeat in a laboratory taste test following negative mood inductions [8]. Some authors suggested that higher scores on self-report EE questionnaires may reflect general eating concerns, rather than increased food consumption in response to negative emotions [9]. In contrast, ecological momentary assessment studies have reported that high self-report EE scores were associated with higher levels of negative affect and lower levels of positive affect before eating occurred in the natural environment [10]. In light of these inconsistencies, it may be useful to assess whether, at the basic attentional level, food stimuli, and particularly palatable high-calorie foods, are processed differently in response to negative emotions among individuals who identify themselves as high on EE.

The role of the human attention system is to prioritize stimuli in the environment for further processing [11]. This selection process is influenced by various motivational, emotional, and attitudinal factors. Given the evolutionary role energy-dense foods play in survival, it is not surprising that studies reported an attentional bias to high-calorie foods among healthy individuals (for reviews see [12, 13]). Abnormal patterns of attention to food have been suggested to contribute to and maintain disordered eating patterns [12, 14, 15]. While some studies showed greater attentional bias to food stimuli (i.e., when attention is drawn toward food stimuli) among those with disordered eating, others reported an attentional avoidance (i.e., when attention is drawn away from food stimuli; for review see [12]). It has been suggested that these opposite patterns of attention toward vs. away from food may reflect a conflict between the desire to eat, and, at the same time, pursuit of dieting goals [12].

With respect to attentional biases to food among those high on EE, studies have also demonstrated inconsistencies. While some studies demonstrated an attentional bias to food among those who are high on EE, others showed attentional avoidance or did not show any difference in attention to food among individuals who are high versus low on EE [12]. For example, Husted et al. [16] reported that higher levels of EE were related to slower responses to food pictures, indicating a food-target avoidance according to the authors. In contrast, another study showed increased attentional capture by food cues among women with high versus low levels of EE, as measured using eye-tracking [17].

Two potential limitations in previous studies on attention to food as a function of EE levels are noteworthy. First, most previous studies on attentional biases among those with EE did not account for negative affect. If negative affect is important in modulating eating behaviors among those with EE, then the experience of negative emotions should modulate attention toward or away from food stimuli in these individuals. Second, most studies on attentional biases in individuals with EE did not assess the potential effect of attentional biases on interference control. Interference control allows attending, selecting and responding to a target stimulus while suppressing attention to task-irrelevant stimuli in the environment [18]. Attentional bias towards specific stimuli such as food, involves the allocation of attentional resources to process these stimuli. However, if one’s goal is to suppress attention to these stimuli, interference control would be required to handle this conflict. In daily situations, individuals may need to suppress attention to food in order to focus on task-performance. Embedding food and non-food stimuli within an interference control task, would allow understanding how levels of EE can influence the ability to suppress attention to food stimuli.

The ability to suppress attention to food-stimuli as a function of task-demands may be modulated by mood among those with EE. During a negative mood, high-calorie foods may become means to reduce negative emotional reactivity among those with EE, and therefore would be harder to ignore. This hypothesis can be supported by a previous study that showed enhanced food-related attentional bias among individuals who are high on EE following a negative mood induction [19] (although null results were observed in a similar study using eye-tracking measurements [20]). In contrast, in neutral mood settings, it may be easier for those who are high on EE to suppress attention to high-calorie foods compared to individuals with lower levels of EE due to attentional avoidance from food while in a neutral mood [16]. Specifically, Husted et al. [16] showed that individuals with higher levels of EE demonstrate an attentional avoidance pattern while being exposed to food stimuli. It was suggested that attentional avoidance from food among those with EE is due to a negative salience of food stimuli that triggers attempts at avoidance. This idea is supported by findings showing attentional avoidance in response to non-specific negative emotional stimuli among individuals with high levels of EE [21]. Furthermore, restrained eating and disinhibited eating which are highly correlated with EE are also associated with attentional avoidance from food [22–24]. Nevertheless, in the aforementioned studies, mood was not manipulated.

Thus, the goal of the present study was to assess if and how mood modulates the ability to suppress attention to high-calorie food stimuli among individuals with different levels of EE in the framework of an interference control task. Individuals who reported having high or low levels of EE were randomly assigned to a negative versus neutral mood induction group (induced via an autobiographic writing task), following which, they performed a food-flanker task. This task allowed for the assessment of food-interference (i.e., the extent to which food stimuli interfered with performance while participants attended a non-food target) and non-food interference (i.e., the level of interference from non-food stimuli while participants attended a food target), separately. We hypothesized that participants in the high EE group would demonstrate reduced food-related interference compared to those in the low EE group following a neutral mood induction. In contrast, it was expected that following a negative mood induction, those with high EE would present increased food-related interference and reduced non-food interference compared to individuals who are low on EE.

## Methods

### Participants

One hundred students from the University of Haifa were screened for the study. All students participated for course credit or payment. Because population based studies repeatedly show significantly higher levels of emotional eating among females compared to males [25–27], only female participants were recruited for the study. Exclusion criteria included a diagnosis of an eating disorder, or any condition that could interfere with cognitive performance such as an attention deficit hyperactivity disorder (ADHD), history of head trauma, or other neurological disorders. Participants completed the emotional eating subscale in the Dutch Eating Behavior Questionnaire (DEBQ-EE; [7]) for screening purposes. The 40 highest on the DEBQ-EE (*M* = 3.99, *SD* = 0.55, Min = 3) and 40 lowest (*M* = 1.65, *SD* = 0.4, Max = 2.4) were recruited for the study. Participants were randomly assigned to a negative or neutral mood induction group, creating four experimental groups with 20 participants in each group.

A power analysis using G × Power 3.1 [28], based on the effect size reported in a previous study that examined links between EE and food vs. non-food interference using the flanker task (η^2^_p_ = 0.15; Husted et al. [16]), indicated that a sample size of 72 participants allows examination of group differences as a function of mood and interference type at a power > 80% with alpha set at 0.05.

### Procedure

The study was approved by the Department of Psychology IRB committee at the University of Haifa (076/19). Eligible participants were invited for a lab visit. To control the state of hunger, all participants were instructed to refrain from eating 3 h before the visit to control for variance related to hunger level between participants [1, 29]. After signing an informed consent form, all participants completed five self-report questionnaires and then practiced the food-flanker task. Following that, participants completed the mood induction task. Before and after the mood induction, participants rated their current positive and negative emotions, as well as their current state of hunger, on a visual analogue scale (VAS). Finally, participants performed the food-flanker task. The entire procedure took place in a dimly lit, sound-attenuated room. At the end of the experimental session, weight and height were measured using a weight scale and a stadiometer to compute body mass index (BMI).

### Measures

#### Self-report questionnaires

*The Dutch Eating Behaviors Questionnaire* (DEBQ: [7]). The DEBQ contains 33 items and three sub-scales: external eating (refers to overeating in response to external food cues; 10 items), emotional eating (refers to overeating in response to emotional state; 13 items) and restrained eating (refers to restrictive eating patterns; 10 items). Cronbach’s α for the DEBQ in the current study was 0.92 and for the EE subscale it was 0.96.

*The State-Trait Anxiety Inventory* (STAI-T: [30]). We used the trait anxiety scale (STAI-T), a measure of overall anxiety. This is a 20-item measure, where each item is rated on a 4-point scale ranging from 1 to 4. In the current study we used only the trait scale. Cronbach’s alpha value in the current study was 0.86.

*The Beck Depression Inventory-II* (BDI-II: [31]). The BDI-II is a measure of depressive symptoms which includes 21-items, where each item is rated on a 4-point scale ranging from 0 to 3. Scores indicate the level of depression (minimal; 0–13, mild; 14–19, moderate; 20–28, severe; 29–63). The Cronbach’s alpha value for the BDI-II in the current study was 0.88.

*The Difficulties in Emotion Regulation Scale* (DERS: [32]). The DERS is a measure of emotion regulation. The DERS is a 36-item measure, where each item is rated on a 5-point scale ranging from 1 to 5. The items reflect difficulties within the following dimensions of emotion regulation: (1) awareness and understanding of emotions; (2) acceptance of emotions; (3) the ability to engage in goal-directed behavior; (4) lack of emotional clarity; (5) impulse control difficulties, and (6) access to emotion regulation strategies perceived as effective. Cronbach’s alpha value for the DERS in the current study was 0.92.

#### The food-flanker task

**Fig. 1:**
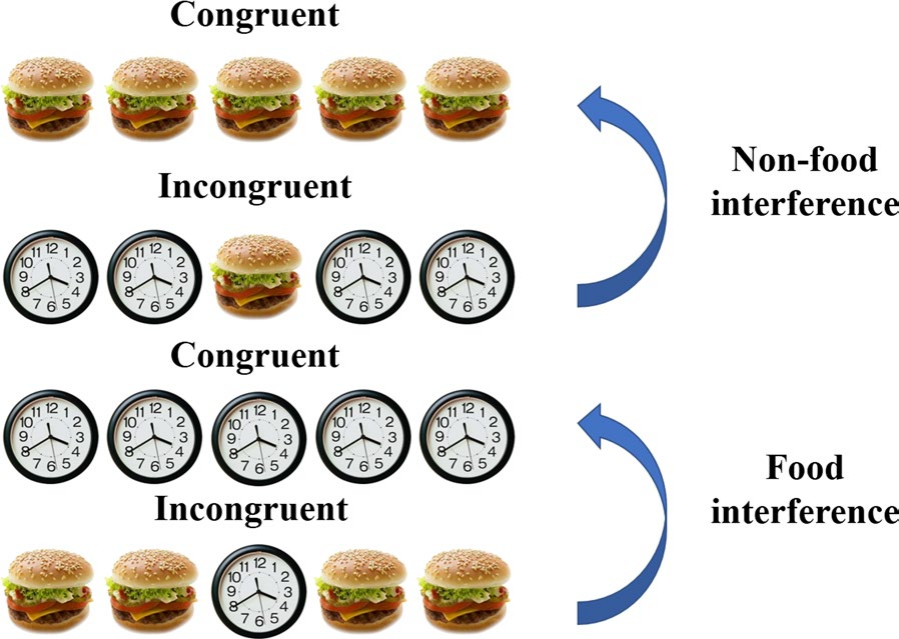
Examples of the different conditions in the food-flanker task

The flanker task is a forced-choice reaction task in which participants are required to respond to a central target stimulus and ignore irrelevant flankers in close proximity to the target [33]. The central target and the flankers compete over a different motor response. In the food-flanker task used for the current study, participants were presented with a fixation cross for 500 ms. Then, a target image appeared at the center of the screen for 2000 ms or until response followed by an inter-trial interval of a blank screen. Participants had to decide via keyboard press, as fast as possible, if the central image depicts food or a non-food item (e.g., press “Z” for food and “M” for non-food stimuli). Four flankers appeared in close proximity to the target (two on each side, see Fig. 1). The flankers were either congruent with respect to the target (e.g., a picture of a non-food image in the center of the screen flanked by the same non-food images), or incongruent (e.g., a picture of a non-food image in the center of the screen flanked by food images). Figure 1 shows examples of the different task conditions. The differences in error rates and response times (RTs) between the incongruent and congruent conditions (i.e., the interference effect) represent the participant's ability to focus on a target and ignore irrelevant distracting information. Larger interference effects represent greater distractibility. The food-flanker task that was used in the current study allowed us to calculate food and non-food interference effects separately (see Fig. 1). The images in the task included 20 palatable food images (with an equal proportion of sweet and savory foods) and 20 non-food images (household items), taken from a food pictures database [34]. Overall, the task included two blocks, each containing 80 trials resulting in a total of 160 trials. There was an equal proportion of food-target and non-food-target trials in a block. The order of trials was randomized. Between blocks, participants were allowed to take a short break. Prior to the task, participants performed 16 trials of practice in which feedback was given in case of an erroneous response.

#### Biographic recall mood-induction task

In order to induce negative/neutral mood, the current study used a written biographic recall mood-induction task [35–38]. In contrast with other mood induction techniques (such as emotional movies or pictures), this method is an idiosyncratic emotion elicitation technique that uses each participant’s unique definition of “neutral” or “negative” mood. In addition, autobiographic mood induction has been successfully used in previous research [39], including in overweight women [40], and in those with high levels of EE [20]. Participants in the negative-mood condition were asked to write a memory of an event that made them feel negative feelings. They were instructed to write about the course of the event, what negative emotions they experienced at the time of the event and afterward. In the neutral-mood induction group, participants were asked to describe in detail a typical, mundane, routine day in their lives, including describing all actions from morning to evening. Both groups were given 4 min to write the biographic memory, and another 2 min of waiting, so they could contemplate the event.

### Visual analogue scale (VAS)

In order to assess the success of the mood induction task, participants were asked to rate their current negative/positive affect as well as hunger level on a VAS before and after the mood induction. Specifically, participants were asked to mark on the VAS the extent to which they currently feel: happy, sad, angry, excited, relaxed, proud, afraid, guilty, and ashamed (running from 0 to 100). The words were taken from the positive and negative affect schedule (PANAS; Watson et al. [41]). The words ‘hungry’ and ‘satiated’ were inserted among the other items in order to make sure that baseline hunger level was equal between the groups.

### Statistical analyses

Demographic and clinical characteristics were analyzed using independent sample t-tests to assess potential differences between the high and low EE groups. To perform a manipulation check, two mixed model analyses of variance (ANOVA) were conducted using rating of negative emotions as the dependent variable in one analysis and positive emotion in the other. Independent variables of these analyses were mood induction (negative\neutral) as an independent between-subject variable and time (before\after the mood induction) as a within-subject independent variable. To examine the main hypothesis in the food-flanker task, a mixed model ANOVA with the interference effect (incongruent RTs minus congruent RTs) used as the dependent measure, EE group (high\low) and mood induction (negative\neutral) as independent between-subject variables, and interference type (food\non-food) as an independent within-subject variable. Significant effects were further analyzed using planned comparisons. Outliers for the RT analysis included extremely low (< 200 ms) or high (1500 >) RTs. These consisted only 1.2% of the total trials. Furthermore, the RT analysis was conducted only on correct responses to the target. Erroneous responses were not analyzed as these consisted 4.45% of the total trials. This is not surprising considering that the task required simple discrimination between food and non-food stimuli. A post-hoc linear regression analysis to assess the independent contribution of food and non-food interference effects to EE as a continuous variable was conducted as will be described below.

## Results

### Group differences on demographics and clinical characteristics

As shown in Table 1, women who were high on EE displayed significantly higher levels of anxiety (STAI-T) and depression (BDI-II), and had higher BMI relative to those with low EE. There was no difference between the groups in baseline hunger level. There was no difference between the groups in the total level of emotion regulation difficulties (DERS). However, the high EE group had greater difficulties in the clarity and acceptance of emotions subscales in the DERS compared to the low EE group.

**Table 1:** Means and standard deviations (in parenthesis) of demographic and clinical characteristics

	High EE (N = 40)	Low EE (N = 40)	*p* value	Cohen’s d
Age (years)	24.05 (4.59)	23.75 (4.21)	.76	0.06
BMI	25.08 (4.80)	22.98 (4.58)	.04	0.44
STAI-T	2.34 (0.56)	2.07 (0.39)	.01	0.54
BDI-II	0.70 (0.54)	0.40 (0.29)	.002	0.70
DERS-total	2.48 (0.69)	2.29 (0.57)	.20	0.28
DERS-acceptance	2.42 (0.96)	1.84 (0.89)	.006	0.62
DERS-clarity	2.31 (0.92)	1.81 (0.75)	.009	0.59
DERS-impulse	2.31 (0.82)	2.42 (0.89)	.58	− 0.12
DERS-awareness	2.40 (0.74)	2.33 (0.85)	.69	0.08
DERS-goal	3.04 (0.94)	3.04 (0.98)	.98	0.04
DERS-strategies	2.59 (0.84)	2.35 (0.84)	.20	0.28
DEBQ-restrained	2.77 (1)	2.21 (0.81)	.008	0.61
DEBQ-external	3.65 (0.49)	3.08 (0.69)	.001	0.94
Baseline hunger level	46.91 (11.14)	46.42 (14.89)	.86	0.03

*EE* emotional eating, *BMI* body mass index, *STAI-T* trait anxiety from the state-trait anxiety inventory, *BDI-II* beck depression inventory-II, *DERS* difficulties in emotion regulation scale, *DEBQ* the Dutch eating behaviors questionnaire

### Manipulation check

The ANOVA using negative emotions’ rating as the dependent variable showed a significant interaction between time and mood condition, *F*(1, 77) = 11.40, *p* = 0.001, η^2^_p_ = 0.12. Planned comparisons revealed that before the mood induction, there was no difference in negative emotions between the neutral (*M* = 12.27, *SD* = 13.78) and the negative (*M* = 14.34, *SD* = 16.27) mood induction group, *F*(1, 77) = 0.371, *p* = 0.54, η^2^_p_ = 0.004, while after the mood induction participants in the negative mood condition (*M* = 27.30, *SD* = 22.19) showed more negative emotions than participants in the neutral mood condition (*M* = 13.08, *SD* = 17.89), *F*(1, 77) = 9.79, *p* < 0.001, η^2^_p_ = 0.11. A similar ANOVA was carried out with the mean rating of positive emotions as the dependent measure. The analysis showed a significant interaction between time and mood condition, *F*(1, 77) = 16.58, *p* < 0.001, η^2^_p_ = 0.177. Specifically, before the mood induction there was no difference in positive emotions between the neutral (*M* = 60.55, *SD* = 21.64) and the negative (*M* = 53.39, *SD* = 21.10) mood induction group, *F*(1, 77) = 2.21, *p* = 0.141, η^2^_p_ = 0.02, wherein after the mood induction participants in the negative mood condition (*M* = 35.01, *SD* = 23.33) showed less positive emotions than participants in the neutral mood condition (*M* = 57.08, *SD* = 24.76), *F*(1, 77) = 16.62, *p* < 0.001, η^2^_p_ = 0.17.

### Results of the food-flanker task

The ANOVA using interference effect (incongruent RTs minus congruent RTs) as the dependent measure (raw RTs are presented in Additional file 1: Appendix 1) and EE group (high\low), mood induction (negative\neutral), and interference type (food\non-food) as independent variables showed a significant main effect for interference type, *F*(1, 76) = 11.83, *p* < 0.001, η^2^_p_ = 0.135. The interference effect was larger for food flanker trials compared to non-food flanker trials. No significant main effects for mood, *F*(1, 76) = 0.05, *p* = 0.82, η^2^_p_ = 0.0006, or EE group, *F*(1, 76) = 0.03, *p* = 0.84, η^2^_p_ = 0.0004, were found.

**Fig. 2:**
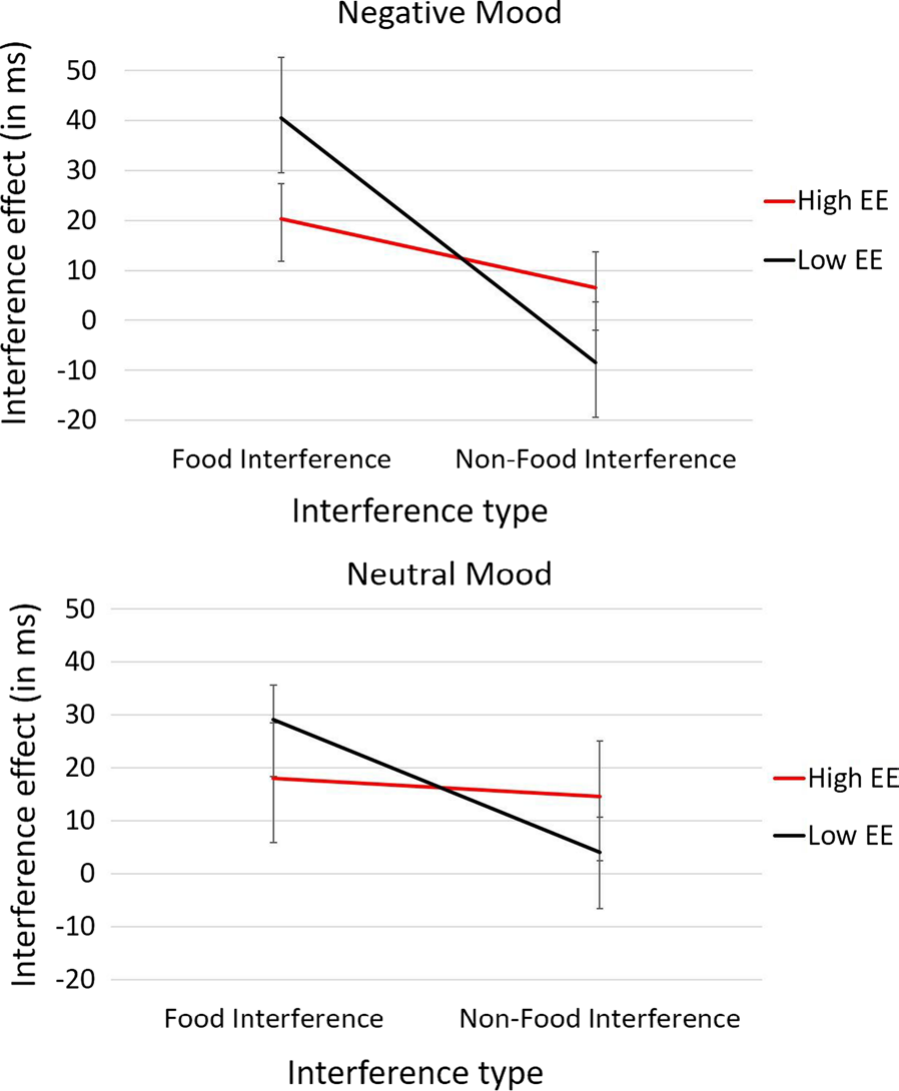
The y-axis represents the mean interference effect (in ms). The upper graph shows the interaction between emotional eating and interference type in the negative mood induction group and the lower panel in the neutral mood induction group

Contrary to our hypothesis, there was no three-way interaction between mood induction, EE group, and interference type *F*(1, 76) = 0.27, *p* = 0.60, η^2^_p_ = 0.02 (see Fig. 2). Nevertheless, the analysis did reveal a significant interaction between EE groups and interference type, *F*(1, 76) = 4.60, *p* = 0.03, η^2^_p_ = 0.057. The pattern of the interactions showed that for the low EE group, food interference was greater than non-food interference *F*(1, 76) = 18.67, *p* < 0.001, η^2^_p_ = 0.17. In contrast, in the high EE group, there was no difference between food and non-food interference *F*(1, 76) = 0.72, *p* = 0.40, η^2^_p_ = 0.01. No other interaction effect was significant (*p*’s > 0.20).

In order to assess whether the absence of the typical attentional bias to food in the high EE group represents an attentional avoidance from food, we conducted a post-hoc linear regression analysis to assess the independent contribution of food and non-food interference effects to EE as a continuous variable. This regression analysis included EE score as the dependent variable and food and non-food interference effects as the predictors. The regression model was significant, *R*^2^ = 0.08, *F*(2,79) = 3.55, *p* = 0.033. Furthermore, the analysis showed that the beta coefficient for the food interference effect was negative and significant, *β* = − 0.22, *p* = 0.041, indicating that lower food interference was associated with higher scores of EE. Additionally, the beta coefficient for non-food interference was positive and near significance level, *β* = 0.21, p = 0.057, indicating that greater non-food interference was associated with higher scores of EE throughout the sample.

## Discussion

The goal of the current study was to investigate the effect of mood (neutral vs. negative) on food versus non-food interference among females who identify themselves with high or low levels of EE. Our findings demonstrated that participants in the low EE group had greater difficulty focusing on a central target when task-irrelevant food images were presented in close proximity to the target, compared to when non-food images were used as irrelevant distractors. This effect was absent in the high EE group. Additionally, a regression analysis showed that higher levels of EE were associated with greater non-food interference and reduced food interference. Specifically, when the central target was a food stimulus and the flankers were non-food stimuli, higher levels of EE were associated with greater attention to the irrelevant non-food stimuli (i.e., attention away from the food target), thus, resulting in greater non-food interference. In contrast, when the flankers were food stimuli and the central target was a non-food stimulus, higher levels of EE were associated with reduced attention to the flanking food stimuli (i.e., attention away from the food flankers), resulting in reduced food-interference. In contrast to our hypothesis, the negative mood induction did not increase food-related interference among those with high levels of EE and in fact, had no influence on the results.

Results of the current study support previous findings showing that food stimuli attract more attention compared to non-food stimuli in healthy individuals [12, 13, 42, 43]. In the current study, this effect was reflected by increased food compared to non-food interference in the low EE group. These results likely reflect the evolutionary importance of high-calorie foods to human survival; a selective detection of energy-dense foods is one of the most adaptive characteristics of human beings [44], making such foods important targets for the attention system. Also, in western food-rich societies, high-calorie foods are promoted excessively as desirable and accessible [45]. This may also exacerbate the difficulty to ignore these types of foods when present, as was shown in the current study.

The results also demonstrate a unique pattern of attention to food among those who reported high levels of EE. The lack of the typical attentional capture of high-calorie foods in the high EE group may reflect a food-avoidance attentional tendency as was also suggested in a previous study on EE [16]. The regression analysis provided support for this notion by demonstrating that increased levels of EE were associated with a greater tendency to divert attention away from food stimuli, which was reflected by increased flanker interference when the target was a food stimulus and the flankers were non-food stimuli and reduced flanker interference when the flankers were food stimuli and the target was a non-food item. Diverting attention away from food stimuli may be a general marker of disordered eating since similar findings were observed in other types of eating behaviors and disorders. For example, attentional avoidance from food stimuli was also reported among individuals with high levels of restrained eating [22, 23] and unsuccessful dieters [24] which are highly correlated with EE [7, 24, 46]. Additionally, a recent study demonstrated that while adolescents without anorexia nervosa display a significant attention bias to food cues, adolescents with anorexia nervosa did not. It was suggested that patients with anorexia nervosa lack the typical bias involved in healthy eating behavior [47].

In contrast to our hypothesis, the negative mood induction did not increase food-related interference among participants with high levels of EE. The fact that mood did not influence attention to food in these participants supports several previous studies, suggesting that those who identify themselves with high levels of EE do not seem to overeat after a negative mood induction compared to other conditions [8, 9, 48]. While these studies focused on eating behaviors per se, the current study aimed to assess performance of more basic attentional mechanisms that operate prior to the behavioral outcome. Nevertheless, the negative mood induction had no impact on attention to food stimuli. Thus, the current findings could indicate that negative affect does not contribute strongly to EE and poses a question regarding whether individuals who identify themselves with high levels of EE actually present different attentional and behavioral reactions to food while experiencing negative emotions. A previous study found that unhealthy snack consumption is not predicted by self-reported EE but depended on the habit of unhealthy snacking and that high scores of EE reflect a fixation on negative aspects of eating [49]. The authors argued that EE does not necessarily represent the tendency to eat under emotional conditions, but rather reflects increased concerns about eating (i.e., negative emotions are a consequence of eating behavior, and not the other way around). Our study may support this assumption since negative emotions did not modulate interference control in the presence of food among those with high EE. Nevertheless, the interpretation regarding the lack of aberrant reactions to food following a negative mood induction among individuals with high EE should be viewed under the potential limitations of our study.

Although the mood induction manipulation used in the current study successfully elevated negative emotions and reduced positive emotions immediately after the mood induction, it could be that this effect was short lived and therefore did not influence performance in the food-flanker task. It could be that a manipulation triggering negative emotions on a trial-by-trial basis could provide different results. It is also plausible that individuals with EE tend to overeat in response to specific emotions. In the current study, we could not control the type of emotion elicited by the autobiographic writing manipulation. Altheimer and Urry [50] recently suggested that those who identify themselves with high levels of EE may not eat following any emotion, but only after emotions that they have learned to associate with eating in the past. Thus, in future research, it might also be useful to use idiosyncratic mood inductions that target the specific negative emotion which triggers different attentional tendencies in response to food compared to non-food stimuli for every individual participant. Another limitation of the present study is that most of the participants were female university students, which may limit the ability to generalize our results to other populations. Further, because individuals who are high on emotional eating are often higher on other types of disordered eating behaviors such as restrained eating and external eating [21, 51, 52], it is possible that other disordered eating behaviors may have contributed to the reported effects. Lastly, actual food intake was not assessed in the current study, therefore it is difficult to assess how the attentional patterns observed translate into actual eating behaviors.

## Conclusions

Overall, this study adds to the growing body of research examining the relationship between EE, mood and interference by food stimuli. The study showed that there is a difference between women who reported having high or low levels of EE in their ability to use interference control while being exposed to food stimuli. In contrast to those with high EE, who were attracted to and interfered by food stimuli, the performance of those with high EE was not interfered by food stimuli to the same extent because higher levels of EE were associated with smaller food-related interference. We suggested that these results reflect a food-avoidance pattern among those with high levels of EE that is due to a broader avoidant strategy that is activated in response to emotionally negative content. The extent to which negative emotions influence this food-avoidance reaction should be clarified in further research.

## Supplementary Information

**Additional file 1**.** Appendix 1. Table S1**. Mean response times (in ms) and standard errors for each experimental condition.

## Data Availability

The datasets used for the current study are available from the corresponding author on request.

## Abbreviations

EE: Emotional eating
DEBQ: Dutch Eating Behavior Questionnaire
STAI-T: Tait subscale of the State-Trait Anxiety Inventory
BDI-II: Beck Depression Inventory-II
DERS: Difficulties in Emotion Regulation Scale
PANAS: Positive and negative affect schedule
VAS: Visual analogue scale
ANOVA: Analysis of variance
RTs: Response times
BMI: Body mass index
