# Supplementary material for: The effect of mood on food versus non-food interference among females who are high and low on emotional eating
Source: J Eat Disord. 2021 Oct 29;9:140. doi: 10.1186/s40337-021-00497-3 (PMC8555330; doi:10.1186/s40337-021-00497-3)
Supplement: Supplementary file 1 — Additional file 1. Appendix 1. Table S1. Mean response times (in ms) and standard errors for each experimental condition. [file 40337_2021_497_MOESM1_ESM.docx]

Appendix 1

| **Table S1. Mean response times (in ms) and standard errors for each experimental condition.** | | | | | | | | | | | | |  |
| --- | --- | --- | --- | --- | --- | --- | --- | --- | --- | --- | --- | --- | --- |
| **Target** | **Congruency** | | | **EE** | | | **Mood** | **Mean RT** | | | **SE** | |  |
| Food |  | Congruent |  | | High |  | Neutral |  | 623 |  | 27 |  | |
|  |  |  |  | |  |  | Negative |  | 577 |  | 17 |  | |
|  |  |  |  | | Low |  | Neutral |  | 609 |  | 12 |  | |
|  |  |  |  | |  |  | Negative |  | 627 |  | 26 |  | |
|  |  | Incongruent |  | | High |  | Neutral |  | 638 |  | 27 |  | |
|  |  |  |  | |  |  | Negative |  | 582 |  | 17 |  | |
|  |  |  |  | | Low |  | Neutral |  | 612 |  | 15 |  | |
|  |  |  |  | |  |  | Negative |  | 617 |  | 23 |  | |
| Non-food |  | Congruent |  | | High |  | Neutral |  | 630 |  | 31 |  | |
|  |  |  |  | |  |  | Negative |  | 585 |  | 16 |  | |
|  |  |  |  | | Low |  | Neutral |  | 590 |  | 14 |  | |
|  |  |  |  | |  |  | Negative |  | 608 |  | 23 |  | |
|  |  | Incongruent |  | | High |  | Neutral |  | 648 |  | 28 |  | |
|  |  |  |  | |  |  | Negative |  | 605 |  | 18 |  | |
|  |  |  |  | | Low |  | Neutral |  | 619 |  | 12 |  | |
|  |  |  |  | |  |  | Negative |  | 646 |  | 22 |  | |

*Note.* EE = Emotional eating; RT = Response times.
